# Mirror Lake

**Published:** 1885-02

**Authors:** Alice P. Adams

**Affiliations:** San Gabriel, Cal.


### MIRROR LAKE.

There are certain excursions made by all tourists who spend any reasonable length of time at Yosemite, and one of these is the drive to Mirror Lake. The average tourist appears to take it for granted that the lake is a broad sheet of water reaching up the valley for miles, and is accordingly disappointed when he finds it is only an expansion of Tenaya Creek, shortly before its confluence with the Merced River.

"No larger than a respectable frog pond!" growled a sturdy Yankee, one day.

"Well, I'm sure," cried his sprightly daughter, "Here, we've taken this long journey to see Mirror Lake, when there's a good deal bigger lake not half a mile from South Jonesville. Let's go back to the hotel, pa, and get ready for our ride to Gla-zhur Point."

The peculiar charm of Mirror Lake lies in its surroundings. Its counterpart in an ordinary locality would be only a tranquil, willow-fringed sheet of water. But while the clear depths of Mirror Lake continue to reveal such marvellous pictures, its shores will be enchanted ground to nature-lovers. The reflections are best seen before sunrise, and each morning carriages leave the hotels in ample season to reach the lake while it is still in shadow. The drive is delightful, going up one side of the river and returning by another road on the opposite shore; but even in summer, the crisp mountain air makes plenty of wraps and carriage robes a necessity at that early hour.

The road is smooth until you near the lake, there it is very rocky, and about June first, when the river is high, it is frequently two or three feet under water.

It was on a cool morning in April that I first saw Mirror Lake. We left the carriages a short distance from the shore and climbed upon a large rock that sloped to the water's edge, a favorite point of view, as I afterward learned. The dense forest encroached on the uttermost limit of the banks, and the air was chill and damp. We were in the shadow of the Half Dome, directly under that rock-marvel rising straight up into the air nearly a mile above us, while in the water lay its mirrored counterpart, Tissaack's proud crest barely escaping the rock on which we stood. Sometimes there is a discernible current near the middle of the lake, but this morning the water was clear and smooth as glass, and mountains and forests were pictured with absolute fidelity.—*Alice P. Adams, San Gabriel, Cal., in February number Vick's Magazine.*

